# Physiological and Transcriptome Analyses Reveal the Effects of Fertilization on the Yield of Winter Wheat and on the Photosynthetic Performance of Leaves during the Flowering Period

**DOI:** 10.3390/genes15091179

**Published:** 2024-09-08

**Authors:** Lihong Wang, Jia Shi, Hongzhi Zhang, Xunji Chen, Jianfeng Li, Zhong Wang, Xiaorong Li, Xin Gao, Chunsheng Wang, Jianqiang Xia, Zhun Zhao, Yueqiang Zhang, Zheru Fan, Qi Zhao

**Affiliations:** 1 Institute of Nuclear and Biological Technology, Xinjiang Academy of Agricultural Sciences, Urumqi 830091, China; lihongwang@xaas.ac.cn (L.W.); shij@xaas.ac.cn (J.S.); dreamzhz@xaas.ac.cn (H.Z.); chenxj713@163.com (X.C.); hssljf@xaas.ac.cn (J.L.); zhongwang@xaas.ac.cn (Z.W.); lixiaorong@xaas.ac.cn (X.L.); gaoxin@xaas.ac.cn (X.G.); wangchunsheng@xaas.ac.cn (C.W.); xiajianqiang@xaas.ac.cn (J.X.); zhaozhun@xaas.ac.cn (Z.Z.); zherufan@xaas.ac.cn (Z.F.); zhaoqi@xaas.ac.cn (Q.Z.); 2 Key Laboratory of Desert–Oasis Crop Physiology, Ecology and Cultivation, Urumqi 830091, China

**Keywords:** physiological traits, transcriptome, winter wheat, fertilization, photosynthesis

## Abstract

Fertilization significantly affects the growth and development of wheat. However, the precise mechanisms underlying gene regulation during flowering in response to fertilization deficiency remain elusive. In this study, fertilization (F) and non-fertilization (CK) ) treatments were set up to reveal examine the effect of fertilization on the photosynthetic capacity of winter wheat during the flowering period through physiological, biochemical, and transcriptome analyses. Upon analyzing analysing their yield, leaf photosynthetic system exchange parameters during flowering, antioxidant enzyme activity, and endogenous hormone parameters, we found that the F treatment resulted in higher net photosynthetic rates during flowering periods than the CK treatment. The superoxide dismutase (SOD) (83.92%), peroxidase (POD) (150.75%), and catalase (CAT) (22.74%) activities of leaves in treated with F during the flowering period were notably elevated compared to those of CK-treated leaves. Abscisic acid (ABA) (1.86%) and gibberellin acid (GA3) (33.69%) levels were reduced, whereas Auxin auxin (IAA) (98.27%) content was increasedwas increased under F treatment compared to those the results under the CK treatment. The chlorophyll a (32.53%), chlorophyll b (56%), total chlorophyll (37.96%), and carotenoid contents (29.80%) under F treatment were also increased compared to CK., exceeded exceeding those obtained under the CK treatment. Furthermore, transcriptional differences between the F and CK conditions were analyzed, and key genes were screened and validated by using q-PCR. Transcriptome analysis identified 2281 differentially expressed genes (DEGs), with enriched pathways related to photosynthesis and light harvesting. DEGs were subjected to cluster simulation, which revealed that 53 DEGS, both up- and down-regulated, responded to the F treatment. qRT-PCR-based validation confirmed the differential expression of genes associated with carbohydrate transport and metabolism, lipid transport, and signal transduction. This study revealed distinctive transcriptional patterns and crucial gene regulation networks in wheat during flowering under fertilization, providing transcriptomic guidance for the precise regulation of wheat breeding.

## 1. Introduction

Wheat is among the most grown crops globally. The adequate and balanced supply of plant nutrients is of critical importance in improving the productivity of this wheat crops [1,2]. The growth of wheat requires a certain amount of nitrogen [3,4], phosphorus [5], potassium, and micronutrients. Nitrogen fertilizer is a majorsource of nitrogen for proteins and nucleic acids, and its application regulates plant growth. The application of appropriate nitrogen fertilizer quantities at the sowing, jointing, flowering, and grain-filling stages could effectively promote the photosynthetic and other physiological characteristics of winter wheat [6], including the soil–plant analysis development (SPAD) value and the chlorophyll a, chlorophyll b, and carotenoid contents [6] of winter wheat. A reasonable application of phosphorus fertilizer can result in superior grain Zn nutrition quality and simultaneously increase the photosynthesis and leaf area of wheat [7] while concurrently maintaining high production rates and high phosphorus use efficiency [8]. Organic fertilizer increases soil organic carbon stock and soil fertility in wheat fields [9]. The benefits of combining organic and inorganic fertilizers have stimulated our research into the involvement of complex regulatory genes.

Nutritional and reproductive growth are equally importanimportant in the flowering period of wheat. Crop yield, whether vegetative or reproductive, depends on access to an adequate supply of essential mineral nutrients. Higher plants absorb nitrogen through two distinct soil nitrate and ammonium absorption systems: the high-affinity transport system and the low-affinity transport system (LATS) [10]. In the transcriptome analysis of wheat, previous studies have found that the expression levels of genes responsible for carbon and nitrogen metabolism was were significantly higher in wheat varieties with high- nitrogen- use- efficiency than in those with low nitrogen use efficiency [11]. Transcriptome analysis has shown that the differential application of nitrogen alters several pathways in plants, including hormone signal transduction pathways, mitogen-activated protein kinase signallingsignaling pathways, photosynthesis pathways, phenylpropanoid biosynthesis pathways, and ATP-binding and transport protein pathwayss (affecting ATPbinding cassette transporters) [12]. Studies comparing between high and low nitrogen levels have found that dioxygenase-activity-related genes (especially for genes Traes_2DL_D4BCDAA76, Traes_2DL_CE85DC5C0, and Traes_2DL_B5B62EE11) were markedly up-regulated in fast-growing seedlings [13]. Low phosphorus availability can induce activatione of the phosphorus response system in plants, thereby promoting corresponding gene expression and protein synthesis, as well as. This enables us to evaluating evaluate gene transcription and transcriptional regulation in cells at an overall level and quantitatively analyzing analyze changes in gene expression levels under specific conditions [14]. Kaur et al. [15] concluded that up-regulated and down-regulated genes of high-nitrogen-effect varieties were mainly enriched in biological processes such as DNA binding, responses to abiotic stimulus, photosynthesis, carbon fixation, the carbohydrate metabolic process, nitrogen compound metabolic process, nitrate transport, and translation. Differentially expressed genes (DEGs) in wheat seedlings under low levels of phosphorus stress are mainly enriched in via carbon fixation in photosynthetic organs, carbon metabolism in leaves and roots, photosynthesis, glyoxylate, and dicarboxylate metabolism, and interactions between plants and pathogens [16]. In the application of organic and inorganic fertilizer sapplication, three genes, Al818695 (phosphoglycerate mutase), AL821423, and AL821213, are up-regulated in the endosperm of grains [17]. 

Regarding diverse fertilizers, studies on tomato have found that organic soil fertilizer management results in a greater allocation of photosynthetically derived resources to the synthesis of secondary metabolites than to plant growth [18]. Studies on corn have revealed that the combination of nitrogen together with potassium and/or phosphate fertilizers induced more differential genes than does the sole application of nitrogen fertilizer [19]. Transcriptome analyses of nitrogen, phosphorus, and potassium interactions have been performed in sorghum [20], corn [19], and barley [21], while these interactions have been less studied in wheat. Thus, most research has been conducted on single nutrients. Actual wheat production is a multi-step, complex process, and a variety of nutrients are involved in the regulation of wheat growth and development. In light of this, in this study, we conducted fertilization and non-fertilization treatments according to the experimental field soil base values, combined with local high-yield and efficient fertilization schemes. We examined the photosynthetic and physiological effects on wheat leaves during flowering by conducting transcriptome analysis at 5, 10, and 15 d after flowering to unveil key genes and offer insights into biological breeding.

## 2. Materials and Methods

### 2.1. Experimental Site and Cultivar

**Figure 1:**
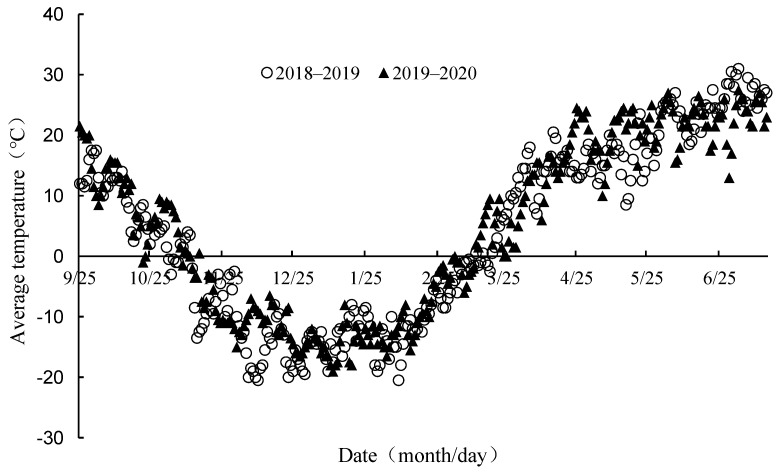
Daily average temperature during the winter wheat growing season in 2018–2019 and 2019–2020.

XinDong 41 (a cultivar that was developed by the Shihezi Agricultural Science Research Institute development and which is officially registered and released by the Xinjiang Crop Cultivar Registration Committee), which is extensively cultivated in Xinjiang, China, was used in this study. The total growth period for this wheat variety, from emergence to maturity, is 272 days. The wheat breeding experimental station of the Xinjiang Academy of Agricultural Sciences in Changji, Xinjiang, China (44°10′ N, 87°44′ E) was regarded as an experimental field in 2018–2020. This region has an altitude of 756 m and a typical continental arid climate, the rainfall during the 2018–2019 wheat growing season was 143mm and that during the 2019–2020 wheat growing season was 167 mm. The specific daily average temperature is shown in Figure 1. The soil at the site is classified as an arenosol in the classification system of the Food and Agriculture Organization (FAO). Table 1 presents the local soil chemical properties (Agricultural Product Quality Inspection Centre, Ministry of Agriculture (Urumqi)).

### 2.2. Experiment Design

The following two treatments were set up in the experiment: CK, without fertilization treatment; F: fertilization treatment, the specific fertilization scheme is presented in Table 2. The F treatments were applied in accordance with the local high standard farmland fertilization guidance programme. Before sowing, fertilizer was sprinkled and turned over thoroughly. During the grain filling period, fertilizer was sprayed by an aircraft, and during other periods, topdressing was applied using water droplets. The total water irrigation amount throughout the growth period was 4950 m^3^ ha^−1^. Water meters with ball valve controls were installed to measure the amount of water provided. The plot area was 275 m^2^ (11 m × 25 m), and the experiment was repeated thrice for each plot. The plants were grown in six rows in a belt configuration with a line spacing of 0.15 m and a sowing density of 270 kg ha^−1^. 

To reduce the error caused by the cultivation environment in the experiment, we ensured that plants subjected to the two treatments were sampled on the same day during the flowering period. We conducted a preliminary experiment and found that the flowering stage under the F conditions occurred approximately 4 days later than that under CK treatment. Therefore, we delayed the CK treatment by 4 days during the suitable broadcasting period. Test materials for F were sown on 25 September 2018 and harvested on 10 July 2019; test materials for CK were sown on 29 September 2018 and harvested on 10 July 2019.

### 2.3. Methods

Flag leaves, which were labelled at the flowering stage on the same day and subjected to the F and CK treatments were examined to assess the blade gas exchange parameters, chlorophyll content, antioxidant enzyme activity, and endogenous hormone content.

#### 2.3.1. Yield and Its Components

At the mature stage, 2 representatives rows of 2 m indoor seed test were obtained from each plot to calculate the grains per hectare, kernels per year and 1000-grain weight. A 4 m^2^ area of each plot was harvested for yield determination. 

#### 2.3.2. Measurement of Blade Gas Exchange Parameters 

Gas exchange (P_n_, T_r_ and C_i_) in wheat flag leaves, which were labelled in the flowering period, was measured using an LI-6400 portable photosynthetic analyzer (LI-COR Biosciences, Lincoln, NE, USA). Measurements were conducted at 30–34 °C, with an ambient CO_2_ concentration of 380–400 μmol mol^−1^ and with relative humidity maintained at 30–32%. Gas exchange parameters were measured from 11:30 to 12:30 h. For each treatment, 5 to 6 leaves were selected, and the average value was calculated.

#### 2.3.3. Chlorophyll Content

During the flowering period, the flag leaves subjected to the F and CK treatments were collected and stored at –20 °C to determine the chlorophyll content of the leaves. Leaf chlorophyll (Chl) was extracted using ethanol and assessed spectrophotometrically following the method described by Lichtenthaler [10]. The chlorophyll a (Chl a), chlorophyll b (Chl b), total chlorophyll, and total carotenoid contents were calculated using the following formulae:

Chl a = 13.95 OD_665_ − 6.88 OD_649_ (1)

Chl b = 24.96 OD_649_ − 7.32 OD_665_ (2)

Car = (1000 OD_470_ − 2.05 Chl a − 114.8 Chl b)/245 (3)

Chl = Chl a + Chl b(4)

#### 2.3.4. Antioxidant Enzyme Activity and Endogenous Hormone Content 

During the flowering period, we selected five representative flag leaves from each treatment, wiped off the dust, quickly placed them in liquid nitrogen, and stored them indoors at −20 °C. We then assessed the antioxidant enzyme activity and endogenous hormone content. Superoxide dismutase (SOD) activity was measured using the nitrotetrazolium chloride blue method [11]. Catalase (CAT) activity was measured according to the method described by Piechowiak et al. [12], and peroxidase (POD) activity was measured using the guaiacol method [13]. Endogenous hormone content was determined by selecting leaves weighing 0.5 g, following the method outlined by Zhang et al. [14].

#### 2.3.5. Transcriptome Sequencing

Flag leaves were collected between 11:00 and 12:00 over three sunny and cloudless days (5th, 10th, and 15th days after flowering) and then promptly frozen in liquid nitrogen. All experiments were performed in triplicate. The material was divided into two parts, one part was used for sequencing, and the other part was used for fluorescence quantification. Both cDNA library preparation and transcriptome sequencing were conducted by Biomarker Technology Co. (Beijing, China). All libraries were sequenced on the Illumina HisSeq 2500 platform (San Diego, CA, USA). Raw data (raw reads) were processed using the NGS QC Toolkit. The reads containing ploy-N and the low-quality reads were removed. Simultaneously, Q30 was calculated. Clean RNA-seq data were obtained, and bioinformatic analysis was performed using the BMKCloud platform (www.biocloud.net, accessed on 8 August 2024).

#### 2.3.6. Validation of DEGs Using Quantitative Reverse Transcription-Polymerase Chain Reaction (qRT-PCR)

SYBR Green qRT-PCR was performed to validate the RNA sequencing data for the 10 DEGs. Wheat actin was used as the internal reference gene, and the LightCycler^®^ 480 II fluorescent quantitative PCR system (Roche Life Science, Basel, Switzerland) and HiScript^®^ II Q RT SuperMix for qPCR (+gDNA wiper) kits (Vazyme Biotech Co., Ltd., Nanjing, Jiangsu, China) were used for detection.

The thermal cycling conditions used for the fluorescence quantitative PCR amplification were as follows: (a) amplification curve: 95 °C for 5 min, followed by 40 cycles of 10 s at 95 °C and 30 s at 60 °C, detection at 72 °C; (b) dissolution curve: 15 s at 95 °C, 60 s at 60 °C, 15 s at 95 °C. The primers used are presented in Table S1. The 2^−△△Ct^ method was used for the relative quantitative analysis of data from the three biological sample replicates. The reactions were performed in triplicates.

### 2.4. Transcriptomic Data Analysis

The raw sequences were transformed into clean reads after data processing and subsequently mapped to a reference genome sequence (https://urgi.versailles.inra.fr/download/iwgsc/IWGSC_RefSeq_Annotations/v2.1/, accessed on 8 August 2024). The expression levels were quantified using Transcripts Per Kilobase of exon model per million mapped reads (TPM). Only reads with a perfect match or single mismatch were further analyzed and annotated based on the reference genome. For each sequencing library, the read count was adjusted using the edge R 4.3.1 package (http://www.r-project.org/, accessed on 8 August 2024) with a scaling normalisation factor. Calculations associated with transcription levels, differential expression analysis, and other applications involved specific tools such as the R package tximport [15], DESeq2, and BiocParallel. The R package pheatmap was used to generate heatmaps, and the Mfuzz R package was used to analyze expression patterns [16]. DEGs underwent Gene Ontology (GO) enrichment analyses via the Triticeae-Gene Tribe platform (http://wheat.cau.edu.cn/TGT/m3/?navbar=Home, accessed on 8 August 2024).

### 2.5. Data Analysis

SPSS 16.0 software (SPSS Institute Inc., Chicago, IL, USA) was used to analyse the data, and Duncan’s new multiple-range test was used to test the difference at the level of 0.05. Results were plotted with Excel.

## 3. Results

### 3.1. Yield and Yield Components

Fertilization enhanced wheat yield. We discovered that the spike number in F-treated plants were 1.13 times and 1.23 times, the grain number per spike were 2.21 and 2.37 times, the thousand-kernel weight were 1.18 and 1.23 times, and the yield were 3.44 and 3.58 times of those in the CK-treated plants in 2018–2019 and 2019–2020, respectively (Table 3). These results showed that the spike number, thousand-kernel weight, yield, and, in particular, the grain number per spike, were significantly increased under F treatment compared to those under CK conditions.

### 3.2. Changes in the Photosynthetic Parameters, Chlorophyll Content, Antioxidant Enzyme Activity, and Endogenous Hormone Content of Wheat Leaves during the Flowering Stage

**Figure 2:**
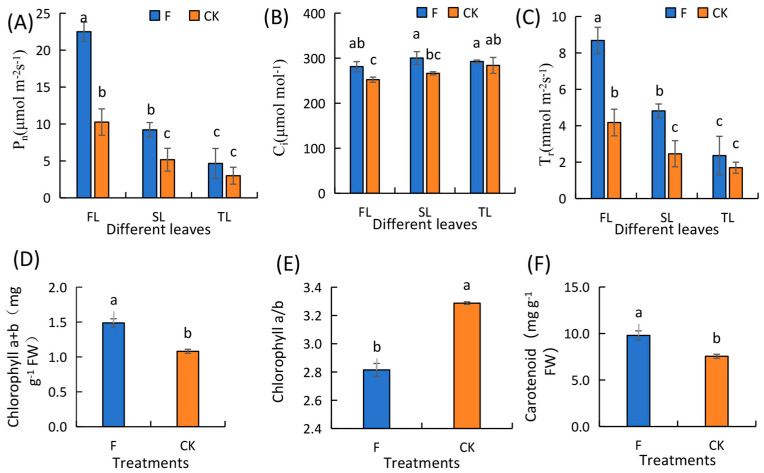
Effects of different treatments on the photosynthetic parameters and chlorophyll content of leaves. (**A**) Net photosynthetic rate (P_n_). (**B**) Intercellular carbon dioxide concentration (C_i_). (**C**) Transpiration rate (T_r_). (**D**) Chlorophyll a and chlorophyll b contents. (**E**) Chlorophyll a/b. (**F**) Carotenoid content. Black horizontal bars represent the LSD at *p* = 0.05 (n = 3); different lower-case letters (“a”, “b” and “c”) denote significant differences at *p* < 0.05 between treatments. CK, without fertilization treatment; F: fertilization treatment. FL, flag leaf; SL, the top second leaf; TL, the top third leaf.

**Figure 3:**
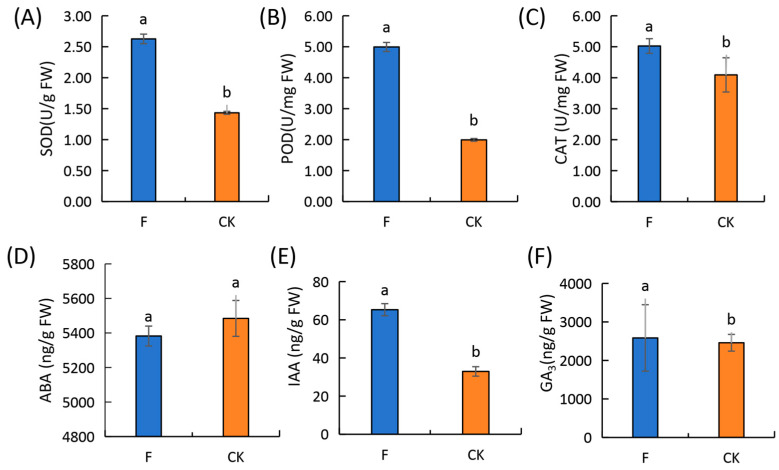
Effects of fertilization on antioxidant enzyme activity and endogenous hormone content in flag leaves of winter wheat during the flowering period (bars show mean and standard error). (**A**) Superoxide dismutase (SOD). (**B**) Peroxidase (POD). (**C**) Catalase (CAT). (**D**) Abscisic acid (ABA). (**E**) Auxin (IAA). (**F**) Gibberellin (GA_3_). Different lower-case letters (“a” and “b”) denote significant differences at *p* < 0.05 between treatments. CK, without fertilization treatment; F: fertilization treatment.

Fertilization significantly affected the photosynthetic parameters and chlorophyll content of wheat leaves. The P_n_ of the flag leaf was higher than that of the second leaf, while that of the second leaf was higher than that of the third leaf at the flowering stage. As leaf position changed to a low level, the differences between plants subjected to F and CK treatments diminished progressively, with no notable disparities observed in the P_n_, C_i_, and T_r_ levels in the third leaf between the treatments. This indicates that fertilization most significantly influences the photosynthetic parameters of wheat flag leaves. Furthermore, an analysis of the flag leaf chlorophyll content revealed that the chlorophyll a+b content, chlorophyll a/b ratio, and carotenoids content of F-treated plants were 1.38 times, 0.85 times and 1.30 times higher than in those under the CK treatment, respectively(Figure 2). Superoxide dismutase (SOD) (83.92%), peroxidase (POD) (150.75%), and catalase (CAT) (22.74%) activities in the leaves of F-treated plants during the flowering period were notably higher than those seen under the CK treatment. Abscisic acid (ABA) (1.86%) and gibberellin acid (GA_3_) (33.69%) levels were lower, while auxin (IAA) (98.27%) content was higher in the F-treated plants than CK-treated plants (Figure 3). 

### 3.3. Characterization of the Sequenced Solexa/Illumina Libraries and Identification of DEGs

**Figure 4:**
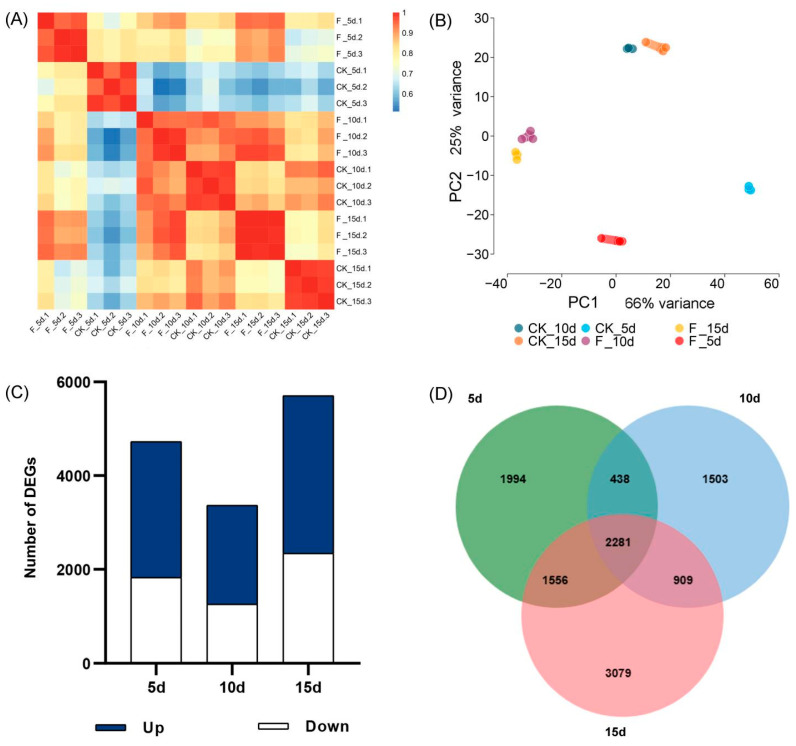
(**A**) Pearson’s correlation analysis between pair-wise samples in each group. (**B**) PCA diagram; (**C**) Differentially expressed genes (DEGs) at three time points; (**D**) Wayne map of DEGs at the three time points (5 d, 10 d, and 15 d). PCA, principal component analysis.

Gene expression exhibited biological variability among individuals. Pearson’s correlation coefficient (r) was used as the evaluation index of the biological repeatability correlation. Pearson’s correlation analysis showed an r^2^ values > 0.9 between each group of samples, indicating that the RNA sequencing data had high biological repeatability and reliability (Figure 4). A comprehensive analysis involving comparative, alternative splicing prediction, and gene structure optimization revealed 2770 new genes, with 13,869 being successfully annotated.

Principal component analysis (PCA) revealed that PCA1 and PCA2 explained 91% of the square difference between the principal components, providing statistical insights into DEGs affecting wheat growth between the F-treated and CK-treated plants on days 5, 10, and 15 after flowering. On day 5, in total 4727 DEGs were identified (*p* < 0.01, | log2 (FC) | ≥ 1), including 2,902 up-regulated and 1,825 down-regulated genes. On day 10, a total of 3367 DEGs were identified (*p* < 0.01, | log2 (FC) | ≥ 1), including 2108 up-regulated and 1259 down-regulated genes. On day 15, a total of 5707 DEGs were identified (*p* < 0.01, | log2 (FC) | ≥ 1), including 3363 up-regulated and 2344 down-regulated genes. The number of DEGs decreased from days 5 to 10 and then increased on day 15. The DEG counts exhibited a decline from day 5 to 10 post-flowering, followed by an increase at the 15-day mark. A total of 2281 DEGs were identified at the three time points, which may play a key role in the response of wheat growth to fertilizer application. 

### 3.4. GO Enrichment Analysis of DEGs

**Figure 5:**
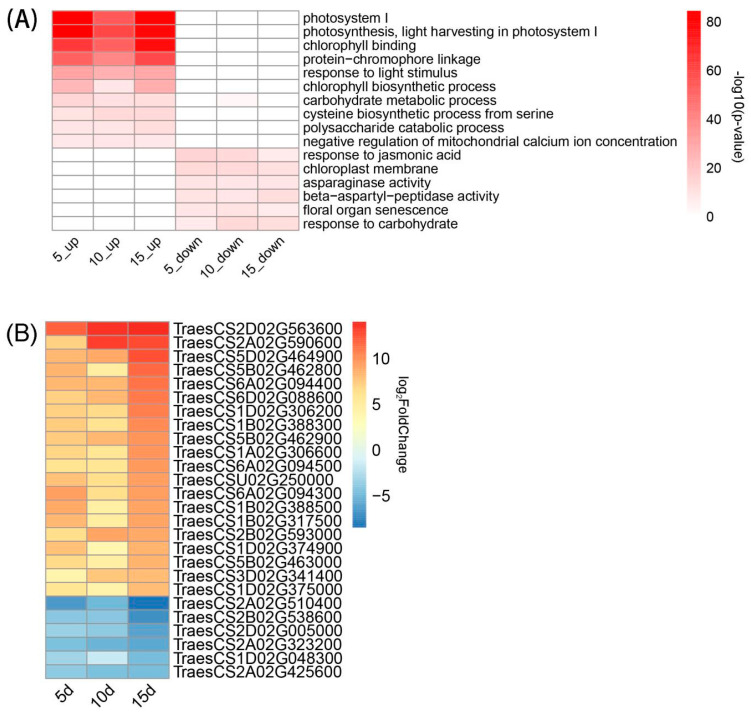
(**A**) Differential expression of GO functions enriched across all three time points between the F and CK treatments; (**B**) Differential multiple sorting of the top 10% significantly different DEGs. DEG, differentially expressed gene; GO, Gene Ontology.

GO analysis was conducted on DEGs at the three time points, and the top 20 GO terms found to be significantly enriched at each point were selected for cross-selection to obtain heat maps of the GO terms significantly enriched at all three points (Figure 5A). At the three time points, significantly up-regulated GO terms were all observed in relation to photosynthesis, encompassing processes such as light harvesting in photosystem I (GO: 0009768, GO: 0009522), chlorophyll binding (GO: 0016168), protein chromophore links (GO: 0018298), and response to light stimuli (GO: 0009416). Conversely, down-regulated GO functions, such as the carbohydrate metabolic process (GO: 0005975), the polysaccharide catabolic process (GO: 000272), and the response to carbohydrates (GO: 0009743), were mainly observed in relation to glucose metabolism, carbon metabolism, and reproductive organ ageing, particularly floral organ sensitivity (GO: 0080187). Fertilizer application affected wheat photosynthesis, leading to the up-regulation of sugar and carbon metabolisms. Without fertilizer, wheat experiences accelerated the ageing of reproductive organs, which highlights the pivotal role of fertilizer in sustaining plant growth and reproductive health.

At all the three time points, there were differences in the genes related to GO terms. In total, 173 and 65 DEGs were up-regulated and down-regulated, respectively. The differential expression multiples of the top 10% of DEGs with the highest differential expression multiples were used for generating the heat maps (Figure 5B). These genes are thought to play key roles in photosynthesis and carbohydrate metabolism.

**Figure 6:**
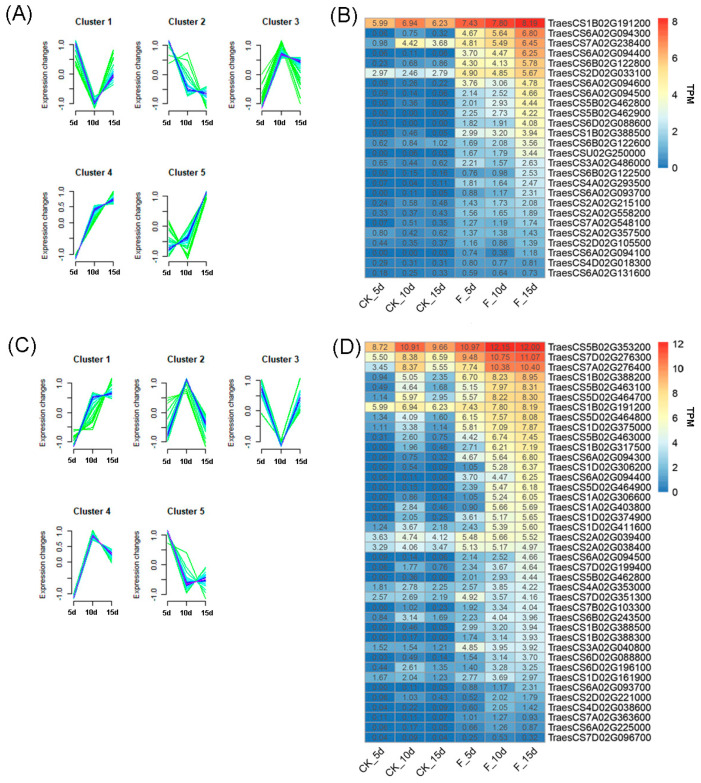
Differences in the expression patterns of up-regulated genes between the fertilizer and CK treatments. (**A**) Up-regulated gene expression patterns in the F group; (**B**) Expression calorimetry of DEGs in cluster 5, which was observed in the F group; (**C**) Up-regulated gene expression patterns in the CK group; (**D**) Expression calorimetry of DEGs in cluster 2, which was observed in the CK group. DEG, differentially expressed gene.

An analysis of the expression patterns of 173 up-regulated DEGs within the selected GO terms revealed cluster 5 exhibited unique expression patterns exclusive to the F treatment, differing from the patterns under the CK treatment (Figure 6A). This cluster comprised 27 DEGs, including 15 chlorophyll a-b binding proteins, 1 glutamyl tRNA reductase 1, 1 α galactosidase 3, 3 glucan endo-1,3-β glucose GI, 1 probable array 5-phase isomerase, one β amylase Tri a 17, 3 basic endochitinases, and one calcium uptake protein. The TPM heat map for each gene is shown in Figure 6B. Figure 6C shows the expression patterns of 173 genes in the control group, with cluster 2 representing the specific expression pattern in the control group, encompassing 40 DEGs. This cluster included 26 chlorophyll a-b binding proteins, 1 α galactosidase, 1 β amylase, 1 β-galactosidase 9, 3 β-glucosidase 16 proteins, 2 chitinase, 1 cysteine synthase, 1 glutamyl tRNA reductase, two oxygen-dependent copinogen-III oxidases, 1 phosphoglycerate mutase-like protein AT74, and one protein G1-like 3. A TPM heat map for each gene is shown in Figure 6D. 

**Figure 7:**
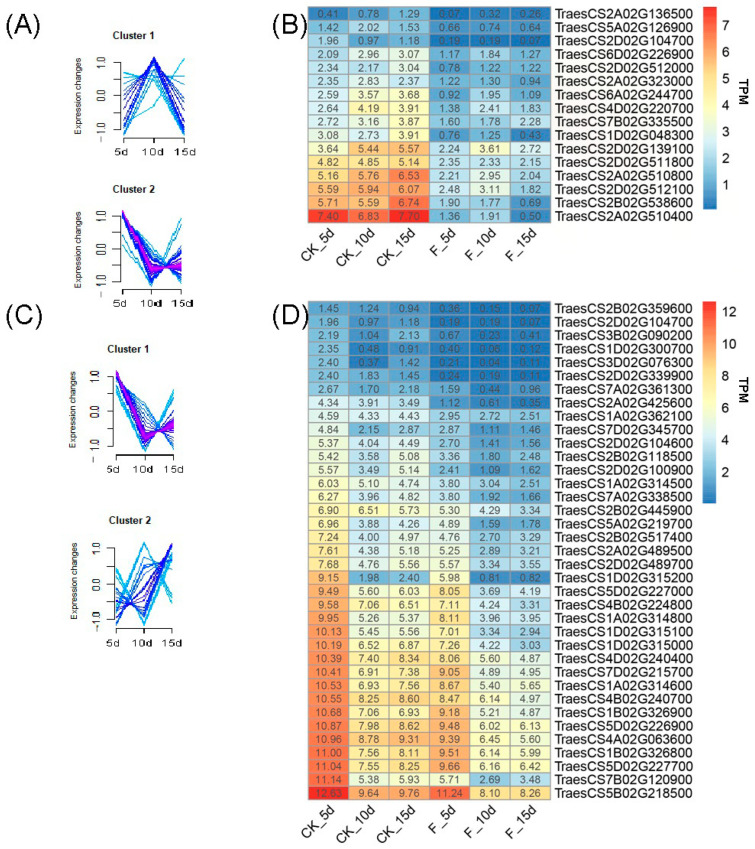
Differences in the expression patterns of down-regulated genes between the F and CK treatments. (**A**) Down-regulated gene expression patterns under the F treatment; (**B**) Expression calorimetry of DEGs in cluster 1, which was observed in the F treatment; (**C**) Down-regulated gene expression patterns under the CK treatment; (**D**) Expression calorimetry of DEGs in cluster 2, which was observed in the CK treatment. DEG, differentially expressed gene.

We analyzed the differences in the expression patterns of 65 down-regulated DEGs (Figure 7A). Among the two expression patterns in the F treatment, cluster 1 displayed unique expression patterns that differed from those seen under the CK treatment. This cluster, including 16 DEGs, involved 2 Tau catenol synthases, two BTB/POZ and TAZ domain-containing proteins, 1 WRKY DNA-binding transcription factor, 1 IAA-amino acid hydroxylase ILR1-like 6, 1 Labd-13Z-ene-9,15,16-triol synthase, 1 protein CHAPERONE-LIKE PROTEIN OF POR1, 1 cationic amino acid transporter 6, 2 purine uracil terms NCS1, and 6 probable isoaspartyl peptides/L-asparaginases 2. The TPM heat map of each gene is shown in Figure 7B. Figure 7C shows the expression patterns of 65 down-regulated genes in the control group, with cluster 2 representing the specific expression pattern in the control group, encompassing 37 DEGs. This cluster included 2 pathogen-associated molecular patterns induced proteins, 8 probable WRKY transcription factors, 2 acyclic sesquiterpene synthases, 2 BTB/POZ and TAZ domains containing proteins, 12 high molecular mass early light inducible proteins HV58, 2 probable folate biopterin transporters 9, 7 low molecular mass early light inducible proteins HV90, and 2 NAC domain-containing proteins 92. A TPM heat map for each gene is shown in Figure 7D. These DEGs may play important roles in down-regulating the differential expression of specific functions.

### 3.5. qRT-PCR-Based Verification of the Expression of Genes Involved in Fertilizer Utilisation Efficiency

**Figure 8:**
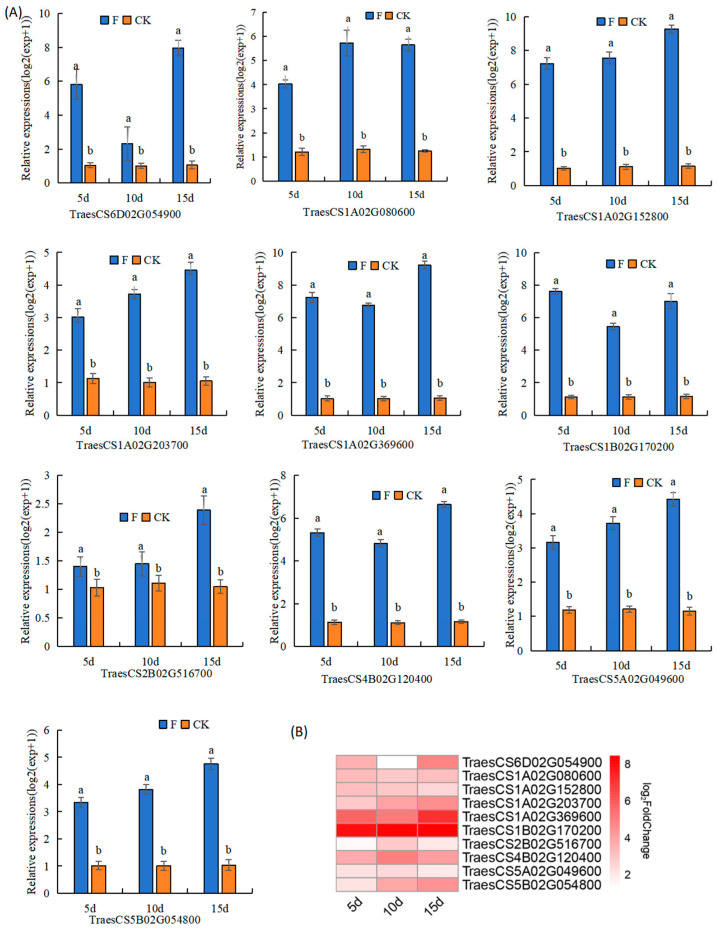
(**A**) qRT-PCR-based validation of the selected genes. The relative expressions levels of unigenes were normalized to those of the internal reference gene, actin. Values represent the mean of three replicates for each sample. Different lower-case letters (“a” and “b”) denote significant differences at *p* < 0.05 among treatments performed on the same day; (**B**) Heat map of log2 fold change for DEGs at 5 d, 10 d, and 15 d for the above 10 genes. DEG, differentially expressed gene; qRT-PCR, quantitative real-time polymerase chain reaction.

Among the DEGs under both F and CK treatments, we identified and selected 10 DEGs with well-defined functional annotations. These were predominantly linked to biological pathways such as photosynthesis and carbon metabolism. These chosen DEGs were subjected to qRT-PCR validation in wheat, with the actin gene serving as an internal reference (Figure 8A). Utilizing the 2^−△△Ct^ method, we conducted relative quantitative analyses of triplicate data from three biological samples, with each reaction replicated thrice. The 10 DEGs under scrutiny, involved in carbohydrate transport metabolism and signal transduction mechanisms, encompassed ethylene reaction factor 1 (ERF1, a pivotal component in ethylene signal transduction-crucial for both biological and abiotic stress responses), RNS1 (secreted ribonuclease), CHI8 (chitinase, playing a role in glycosidic bond hydrolysis on the chitin sugar chain), PEAMT1 (phosphoethanolamine methyltransferase, a key enzyme in betaine synthesis), RNS4 (a member of the ribonuclease T2 protein family), 6-FEHs (fructose exohydrolases), CIPK14 (calcineurin b-like protein interacting protein kinase, playing a negative regulatory role in plant immune response), PIF13 (transcription factor involved in the photosensitive pigment signalling pathway, inhibiting chlorophyll biosynthesis and photosynthesis), PIF3, and POD70 (peroxidase 70). qRT-PCR was used to successfully verify the significant differences in the expression of these 10 genes under the two treatments, and they were consistent with the results of the RNA-seq data (Figure 8B). This result suggests that these genes may play an important role in plant growth under different fertilizers conditions.

## 4. Discussion

### 4.1. Effect of Fertilization on the Physiological Indicators of Winter Wheat Leaves during the Flowering Period

The main winter wheat variety, namely, XD41 from Xinjiang, China, was used as the experimental material in this study owing to its tolerance to fertilizers and water, coupled with its consistently stable yield. The growth of wheat seedlings in field plots is influenced by various environmental factors, including cultivation measures, light, and temperature. In this study, we focused on analyzing the phenotypic and physiological indicators of wheat under fertilizer and non-fertilizer treatments. Certain indicators exhibited significant differences under extreme fertilizer conditions, while others, such as CAT, did not show significant variations, aligning with the results of prior studies [17]. The total photosynthetic pigment content increased with plant age and was higher at higher fertilizer rates. Chl a, Chl b, and carotenoid biosynthesis showed similar responses to nitrogen fertilizer application [18]. In the presence of combined organic manure and inorganic fertilizers, notable improvements were observed in the net photosynthetic rate, flag leaf chlorophyll content, and leaf area index during the mid-grain-filling stage (20 or 23 d post-anthesis). Additionally, these parameters exhibited relatively slower declines at the late grain-filling stage (30 d post-anthesis) compared with those under treatments using only inorganic fertilizers [19]. 

### 4.2. Transcriptome Analysis of the Effect of Fertilization on the Leaves of Winter Wheat at the Flowering Stage

Guo et al. [20] demonstrated that the modified expression of TaCYP78A5 enhances wheat grain weight and grain yield per plant by accumulating auxin. Transcriptome and hormone metabolome analyses revealed that TaCYP78A5 participates in the auxin synthesis pathway and promotes auxin accumulation and cell wall remodelling in the ovary. Additionally, the over expression of TaTPP-7A significantly increased the expression levels of starch synthesis- and senescence-related genes involved in the ABA and ethylene pathways [21]. TraesCS5D02G364100 (chlorophyllase), BGI_novel_G006617 (lycopene epsilon-cyclase), TraesCS4A02G034800 and TraesCS4A02G035100 (Zeaxanthin epoxidase), and TraesCS6B02G122500 (light-harvesting complex II chlorophyll a/b binding protein) were responsible for the degradation of chlorophyll, the synthesis of carotenoid, and light energy harvesting, respectively. The up or down-regulated expression of these genes presumably reduces chlorophyll degradation, increases carotene synthesis, and promotes light energy conversion [22]. Transcriptome analysis revealed that key pathways, such as ‘alanine, aspartate, and glutamate metabolism’, ‘terpenoid backbone biosynthesis’, and ‘vitamin B6 metabolism’ influence nitrogen use efficiency in wheat. The activation of ABA signal transduction and biosynthesis pathways also helps maintain nitrogen use efficiency under low-nitrogen conditions. Moreover, bHLH transcription factors play a positive role in wheat NUE [23]. To further investigate the responses of different genes to fertilization, we conducted a transcriptome sequencing analysis of materials with and without organic and inorganic fertilizer combinations. In total, 2281 DEGs were screened for significant differences at three flowering time points (5, 10, and 15 d), with the results suggesting their importance in wheat fertilizer response. Up-regulated functional terms included those related to photosynthesis, such as light harvesting in photosystem I (GO: 0009768), photosystem I (GO: 0009522), chlorophyll binding (GO: 0016168), protein chromophore junctions (GO: 0018298), and response to light stimulation (GO: 0009416). All these functional terms are all related to wheat photosynthesis [24,25].

The genes exhibited significant enrichment in various metabolic pathways, including carbon fixation, photosynthesis antenna protein, carbon metabolism, amino acid biosynthesis, porphyrin and chlorophyll metabolism, glyoxylate and dicarboxylic acid metabolism, photosynthesis, and glycine, serine, and threonine metabolism. Additionally, autophagy regulation in photosynthetic organisms was observed. Conversely, down-regulated functional terms encompassed carbohydrate metabolism (GO: 0005975), polysaccharide catabolism (GO: 000272), response to carbohydrates (GO: 0009743), and floral organ ageing (GO: 0080187). These terms ware mainly related to substance metabolism, such as sugar and carbon metabolism. Under the non-fertilization treatment, the nutritional utilization efficiency of wheat itself may be improved, as suggested by Zhang et al. [14]. The down-regulation of functional terms related to plant ageing [26,27] indicates a close connection between wheat senescence during the flowering period and nutrient supply. This relationship is evident in production practises, where plants subjected to relatively high fertilizer levels during flowering tend to exhibit a relatively greener appearance, consistent with the findings of this study.

### 4.3. Verification and Regulatory Analysis of Key Genes

Among the terms exhibiting significant expression differences at the three time points, we focused on the top 10% of all DEGs, identifying 27 DEGs with multiple distinctions. These DEGs exhibited the most significant differences between the fertilizer and non-fertilizer treatments. Subsequently, the expression patterns of the DEGs in these significantly different GO terms were simulated using this method. In our screening, we identified 66 up-regulated and 53 down-regulated DEGs. We posit that genes displaying diverse expression patterns in F and CK treatments may serve as key players in responding to fertilizer levels. Notably, among the DEGs screened through both methods, four DEGs are pivotal in the expression mode and exhibit significant differences in expression levels between F and CK treatments, reaching the top 10%: TraesCS1D02G048300, TraesCS2A02G425600, TraesCS2A02G510400, and TraesCS2B02G538600. Among these, two genes encoding asparaginase peptidase/L-asparaginase 2 have been reported to be important enzymes involved in nitrogen assimilation in wheat. These enzymes facilitate the production of asparagine and glutamic acid by transferring amide nitrogen from glutamine to aspartate, participating in multiple biological activities in plants [28]. Subsequent studies delved into the analysis of these two terpenoid synthase genes.

Simultaneously, while examining the expression patterns at three time points, we observed that the expression levels of both up-regulated F treatment expression pattern 1 and up-regulated CK treatment expression pattern 3 initially decreased and then increased. We analyzed the genes clustered in these two expression patterns, mainly focusing on chitinase, glucanase, aspartate protease, serine acetyltransferase, and calcium transporter. Additionally, cluster 1, subjected to fertilization, included genes associated with protein networks. Plant chitinases play a crucial role in degrading chitin within fungal cell walls, reducing the pathogenicity of invasive pathogens. Simultaneously, chitinoligosaccharides generated from chitin degradation act as elicitors, triggering plant defence reactions [29,30]. Dextran endonuclease, which can catalyze the hydrolysis of 1,4- β-polysaccharides in the main chain of pectin, such as cellulose and xyloglucan molecules, participates in the modification of cell walls. Aspartate protease in plants primarily participate in cathepsin D and cathepsin E within lysosomes, playing roles in precursor protein processing, protein degradation, and programmed cell death.

Aspartate proteases are widely distributed in processes such as programmed cell death and are involved in 20 plant diseases, stress responses, and leaf senescence processes [31,32]. These genes are primarily involved in plant defence mechanisms. The expression levels of BTB/POZ and TAZ domain-containing protein, bZIP transcription factor, WRKY DNA-binding transcription factor, and other transcription factors were predominantly observed in the expression patterns of up-regulation without fertilizer and down-regulation with fertilizer. The expression levels of these genes showed a trend of initially decreasing/increasing and then increasing/decreasing. 

In addition, in the CK treatment, the down-regulation of wheat leaf metabolic pathways was mainly related to material metabolism, including the utilization of carbon sources as respiratory substrates, such as starch and sucrose, and the metabolism of other carbohydrates. DEGs were mainly concentrated in two pathways: starch and sucrose metabolism, and glucose metabolism. Key genes within these pathways included 6-FEH, vacuolar invertase, glucose epimerase, cell wall invertase, β- Glucosidase, sucrose phosphate synthase, sucrose synthase, and pectin.

A limitation of this study is that the analysis was performed from the physiological, biochemical, and transcriptomic perspectives, and we focused on selecting 10 genes with significant differences in photosynthetic capacity.

## 5. Conclusions

This study indicates that fertilizer plays a regulatory role in the antioxidant enzyme activity, endogenous hormone activity, and chlorophyll content of flag leaves during wheat flowering. The GO enrichment of DEGs treated with and without fertilizer during flowering revealed that up-regulated pathways were associated with functions such as photosynthesis and light capture, whereas down-regulated pathways were linked to bioenergy metabolism and flower organ senescence. The identification of key genes through cluster screening and the validation of 10 candidate genes via qPCR offers new insights into the underlying mechanisms of the effects of fertilizer on wheat. These findings unveil potential regulatory pathways and candidate genes involved in fertilizer, providing a theoretical basis for further research.

## Figures and Tables

**Table 1:** Soil conditions in the trial plots.

Layer (cm)	Organic Substance (g kg^−1^)	Total Nitrogen (g kg^−1^)	Total Phosphorus (g kg^−1^)	Total Potassium (g kg^−1^)	Available Nitrogen (mg kg^−1^)	Available Potassium (mg kg^−1^)	Available Phosphorus (mg kg^−1^)	Total Salt (g kg^−1^)	pH
0–20	11.35	0.807	1.004	22.078	43.3	14.3	136	0.80	8.76

**Table 2:** Fertilization schemes for different treatments.

Period	F (kg ha^−1^)			CK
	N	P_2_O_5_	K_2_O	
Before sowing	97.5	75	150	/
Reviving	41.4	/	/	/
Erecting	41.4	/	/	/
Jointing	110.4	/	31.2	/
Heading	41.4	/	/	/
Flowering	41.4	/	/	/
Filling	34.5	31.2	20.4	/

The organic fertilizer used before sowing contained 45% organic matter, 1.3% N, 1% P_2_O_5_, and 2% K_2_O,. The fertilizer used after sowing contained urea (N, 46%), diammonium phosphate (64% (N,18%; P_2_O_5_, 46%)), potassium phosphate monobasic (P_2_O_5_, 52%; K_2_O, 34%), and potassium sulphate (K_2_O, 52%).

**Table 3:** Wheat yield and its components **under** different treatments.

Years	Treatments	Spike Number (10^4^ spikes·ha^−1^)	Grain Number per Spike	Thousand Kernel Weight (g)	Grain Yield (kg·ha^−1^)
2018–2019	F	535.67 a	42.18 a	52.39 a	9342.80 a
	CK	474.00 b	19.07 b	44.48 b	2719.40 b
2019–2020	F	585.60 a	44.55 a	50.30 b	9854.50 a
	CK	475.80 b	18.82 b	40.97 b	2751.36 b

The error bars represent standard deviations of the means of values from five independent biological replicates. For each trait, different letters (“a” and “b”) represent significant differences from each other according to a two-way ANOVA after testing using least significant difference (LSD) multiple comparison assessments (ρ < 0.05). CK, without fertilization treatment; F: fertilization treatment.

## Data Availability

The original data contributions presented in the study are included in the article, further inquiries can be directed to the corresponding author. The raw sequencing data are available from the National Genomics Data Centre under the accession number PRJNA1049585. The data can be accessed at: https://submit.ncbi.nlm.nih.gov/subs/bioproject/, accessed on 8 August 2024.

## Supplementary Materials

The following supporting information can be downloaded at: https://www.mdpi.com/article/10.3390/genes15091179/s1, Table S1: The primer sequences used for qRT-PCR of the genes involved in fertilizer utilization.
