# Supplementary material for: Physiological and Transcriptome Analyses Reveal the Effects of Fertilization on the Yield of Winter Wheat and on the Photosynthetic Performance of Leaves during the Flowering Period
Source: Genes (Basel). 2024 Sep 8;15(9):1179. doi: 10.3390/genes15091179 (PMC11430998; doi:10.3390/genes15091179)
Supplement: Supplementary file 1 [file genes-15-01179-s001.zip › genes-3175572-supplementary.pdf]

Table S1: The primer sequences used for qRT-PCR of the genes involved in fertilizer utilization

Gene	Sequence (5′-3′)	Length (bp)
POD70	AAGAGCCGTTTGGTTCAGATAA	112 bp
	CATACGTCGGAGCGACTTACA	
ERF1	GGCTACGAGAAACTTCGGATTG	234 bp
	GGTCGGTGGCACATTGGAT	
RNS1	CCTAACACCGTAGCATCCACA	239 bp
	GGTTCATCCTGCCATCCTTAT	
CHI8	TTGGTTAATCGTGTTCGTGCTT	119 bp
	GGACGTGGCCTTGCTTATCTC	
PEAMT1	ATCAGCAACCGCACAGCA	103 bp
	TCTGAACGAGCGACCGAAC	
RNS4	GCCCACAGAACCTACATACCC	119 bp
	ACCGTCTCACCTGAGGCGTA	
6-FEHs	GTTTCCCGTCTGAACACCTTT	113 bp
	GGATCTCCTCGTCCTATCGTCT	
CIPK14	GTTTGACCTGTCCGGCCTAT	178 bp
	TCCTTCCCTCCTTCCTTGC	
PIF13	GCAGAGGAGAAGGGACAGGA	150 bp
	TCGATGCCAAGAACGAGATG	
PIF3	GTATGTAATTCCAATCCCTCCG	149 bp
	TCCAGCATCGACGCCTTGT	
